# Supplementary material for: The Bayesian superorganism: externalized memories facilitate distributed sampling
Source: J R Soc Interface. 2020 Jun 17;17(167):20190848. doi: 10.1098/rsif.2019.0848 (PMC7328406; doi:10.1098/rsif.2019.0848)

The Bayesian Superorganism: externalised memories facilitate distributed sampling

Supplementary Information

Figure S1. We compared observations of heterogeneous arena exploration in our own ants with data on *Lasius niger* exploration. The number of ants leaving a 50x50cm region on each side is shown on the right. For our *T. albipennis* trajectories, the trajectory is coloured black until it first reaches the virtual 50x50cm boundary and blue thereafter (ants continue exploration rather than being removed as in the *L. niger* data). Not all ants in our data leave this virtual region, and so their final position after 45 minutes is marked instead. Statistics test a null hypothesis of uniform exit numbers on each side. Magnetic north is marked (although NB, local field can be affected by the building etc.)

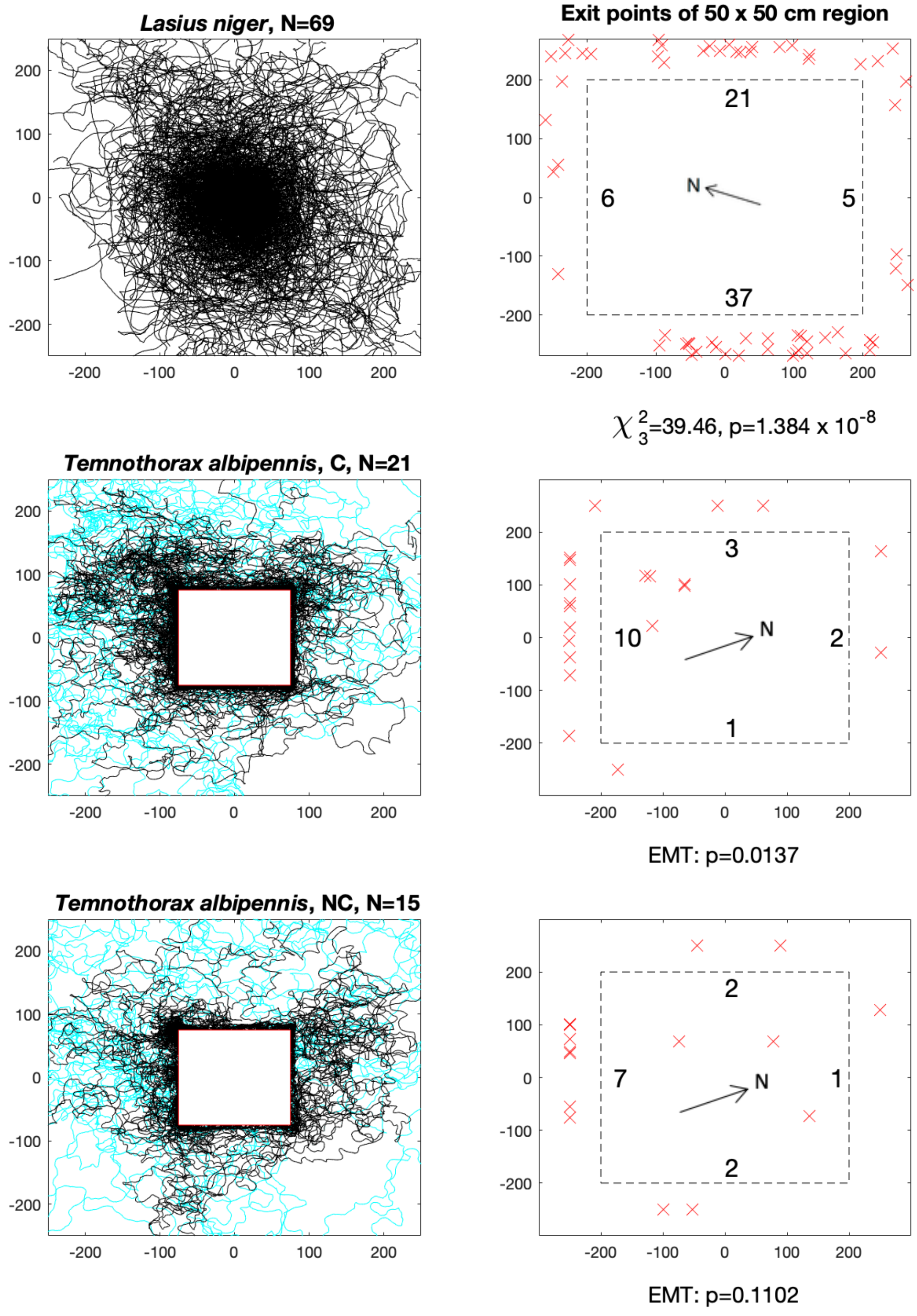

Figure S2. Distribution of ant positions during exploration (histogram bin counts), within a 12.5cm radius circle (i.e. diameter 25cm, half of 50cm virtual arena width), and outside that radius.

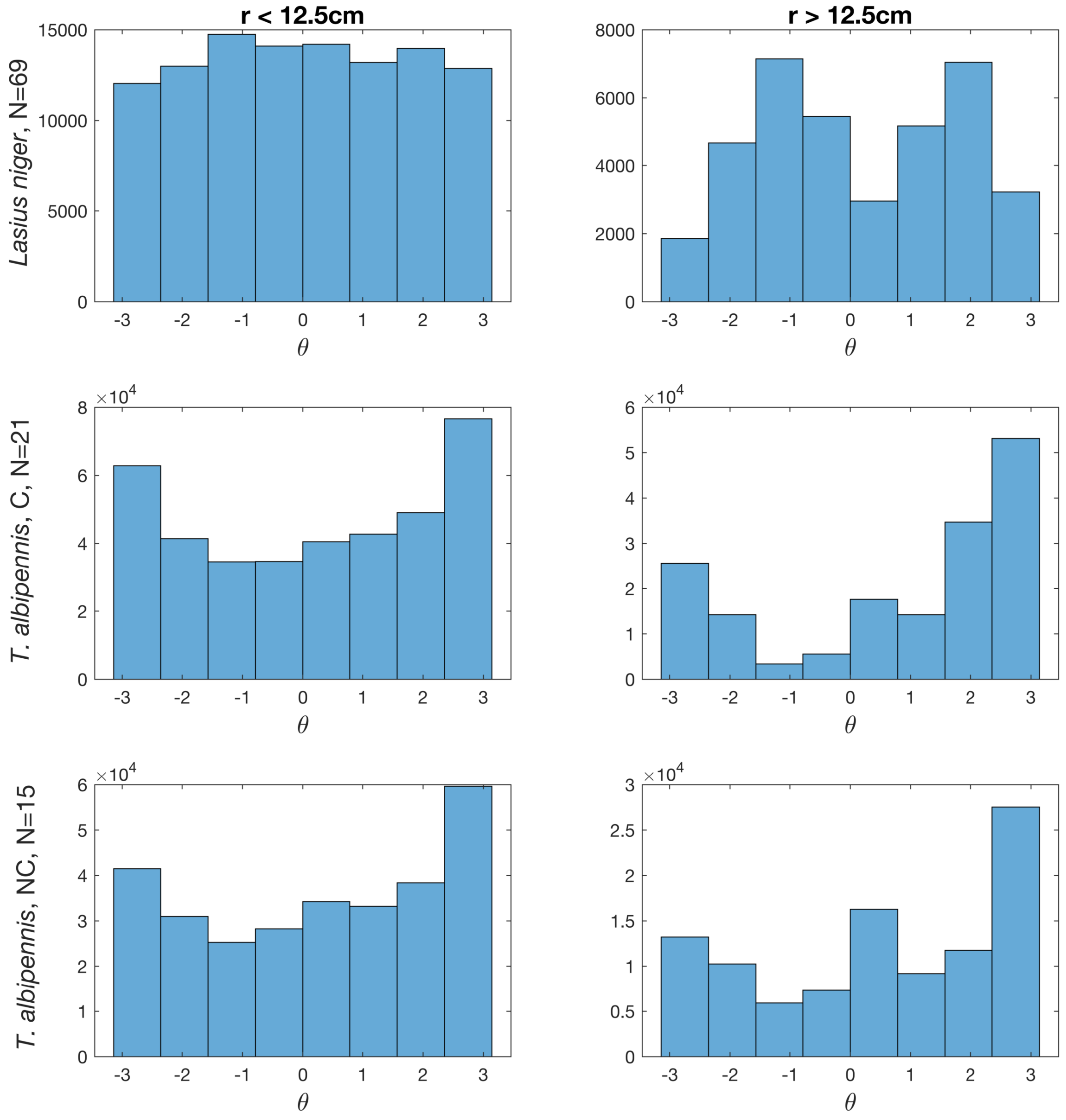

Although the *Lasius niger* trajectories from Khuong et al. uniformly explore the central region, their angular distribution is bimodal outside of it (c.f. Figure 2). The *Temnothorax albipennis* trajectories are somewhat uniform inside the radius (thigmotaxis on the square paper mask affects the distribution), and they are more obviously biased in the $-x$ direction (π radians) outside this area. Note that the NC treatment angular distribution at $r > 12.5$cm is somewhat more uniform than the C treatment, especially around $[-\pi/2, 0]$.

Khuong, A., Lecheval, V., Fournier, R., Blanco, S., Weitz, S., Bezian, J. J., & Gautrais, J. (2013). How do ants make sense of gravity? A Boltzmann walker analysis of Lasius niger trajectories on various inclines. *PloS one*, *8*(10). https://doi.org/10.1371/journal.pone.0076531
Dataset S1, available at: https://doi.org/10.1371/journal.pone.0076531.s005

Figure S3. The individual trajectories of ants in the cleaning (C) treatment.

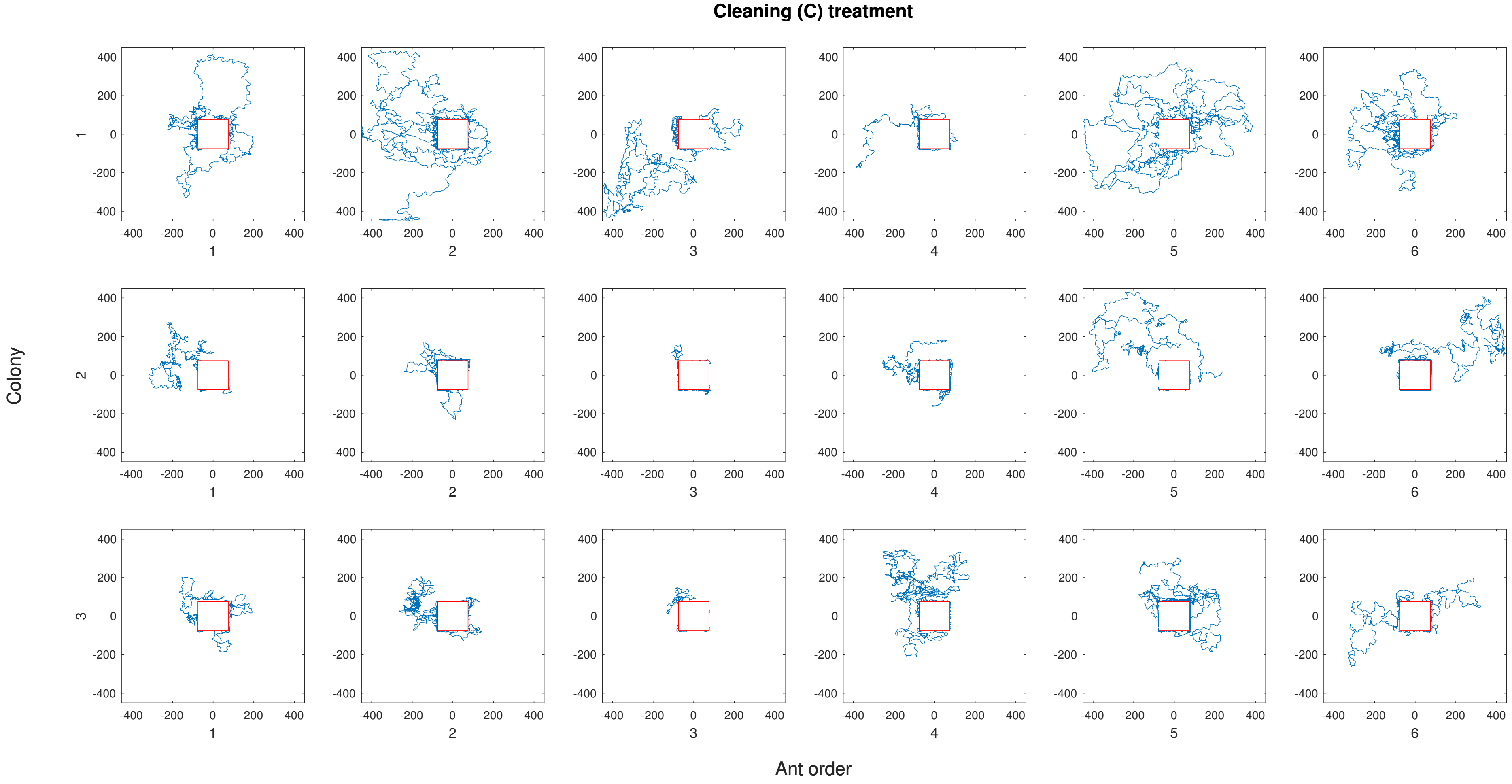

Figure S4. The individual trajectories of ants in the no cleaning (NC) treatment.

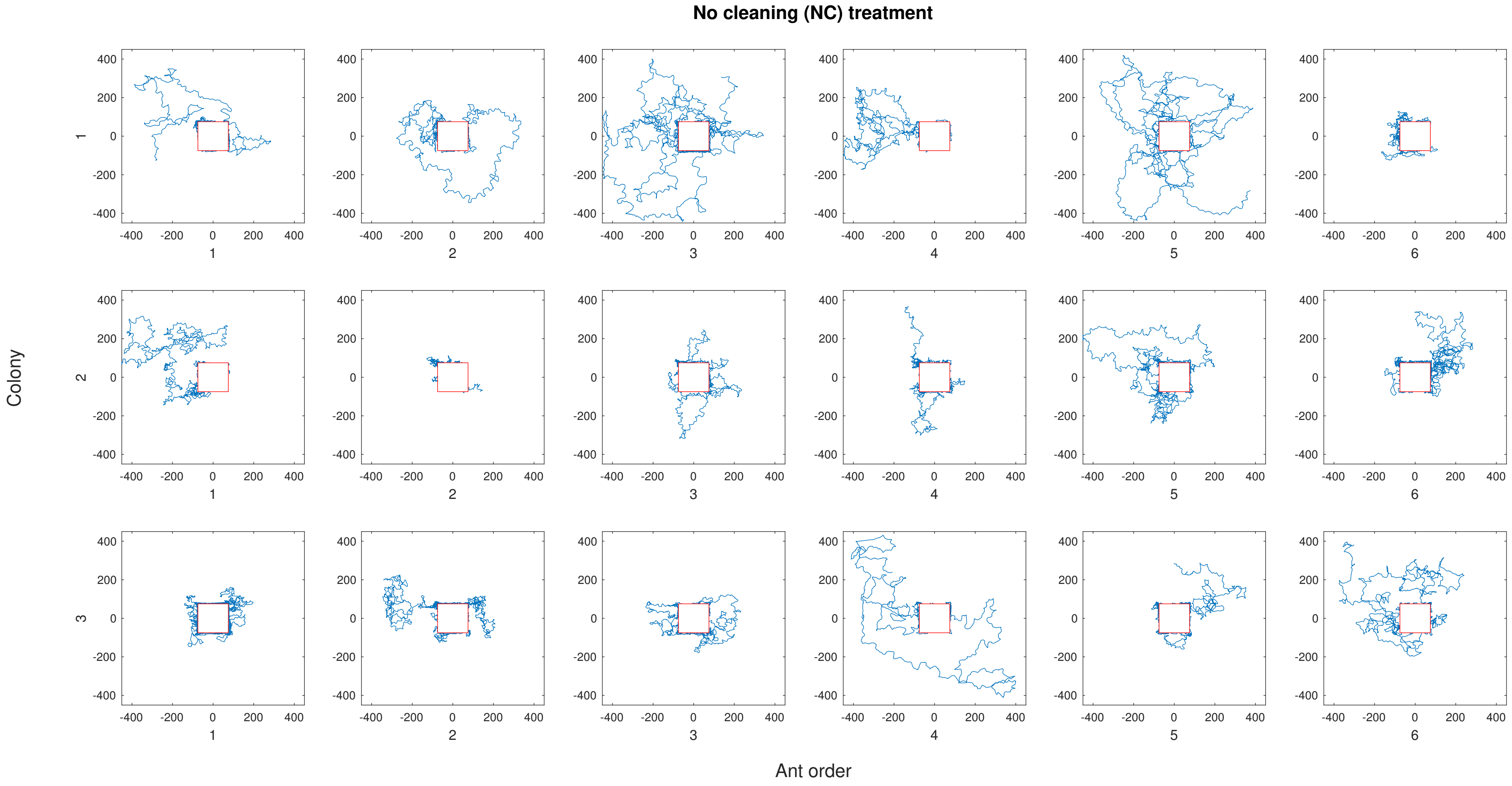

Figure S5. The cumulative trajectories of ants in the cleaning (C) treatment.

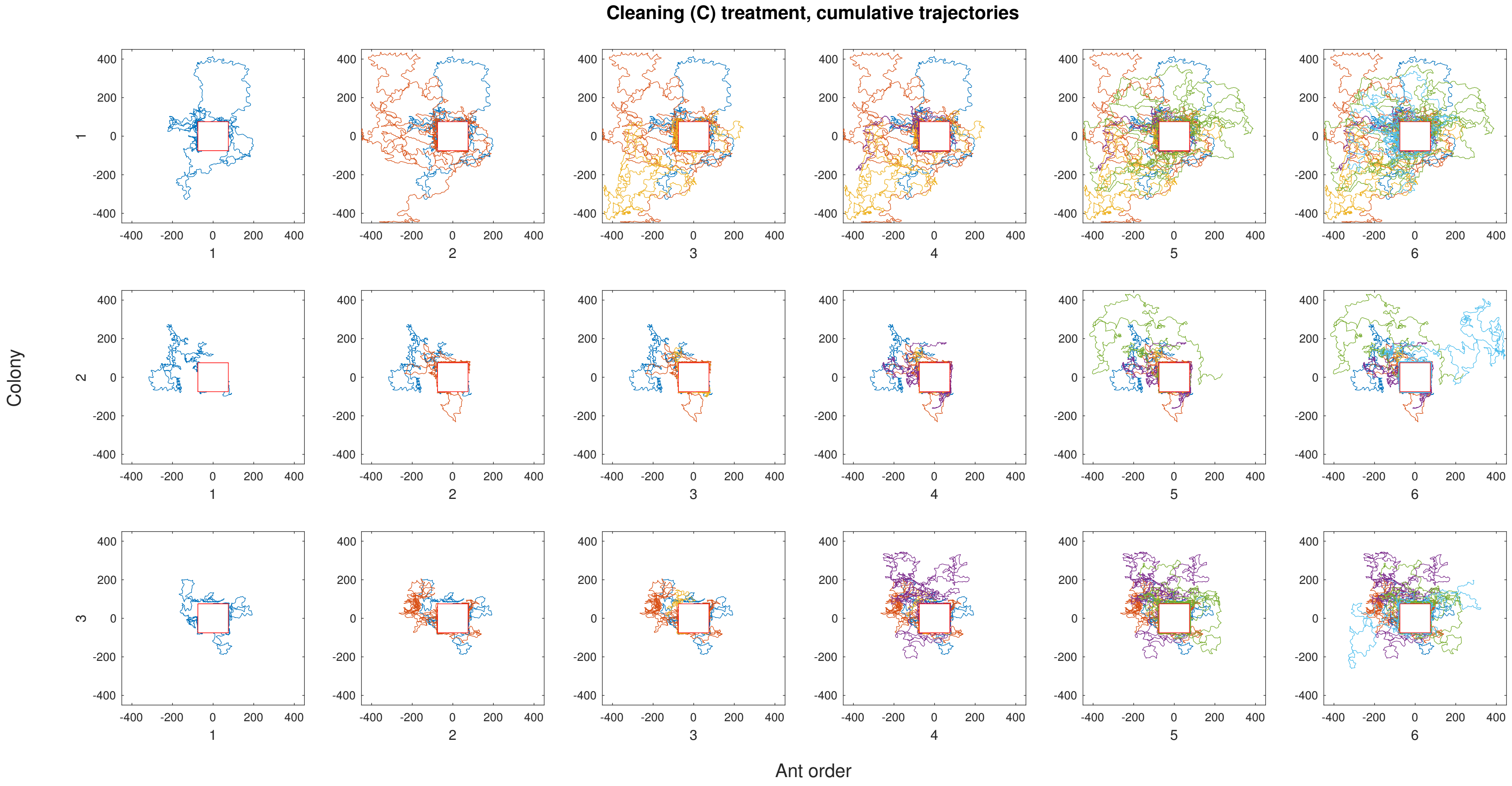

Figure S6. The cumulative trajectories of ants in the no cleaning (NC) treatment.

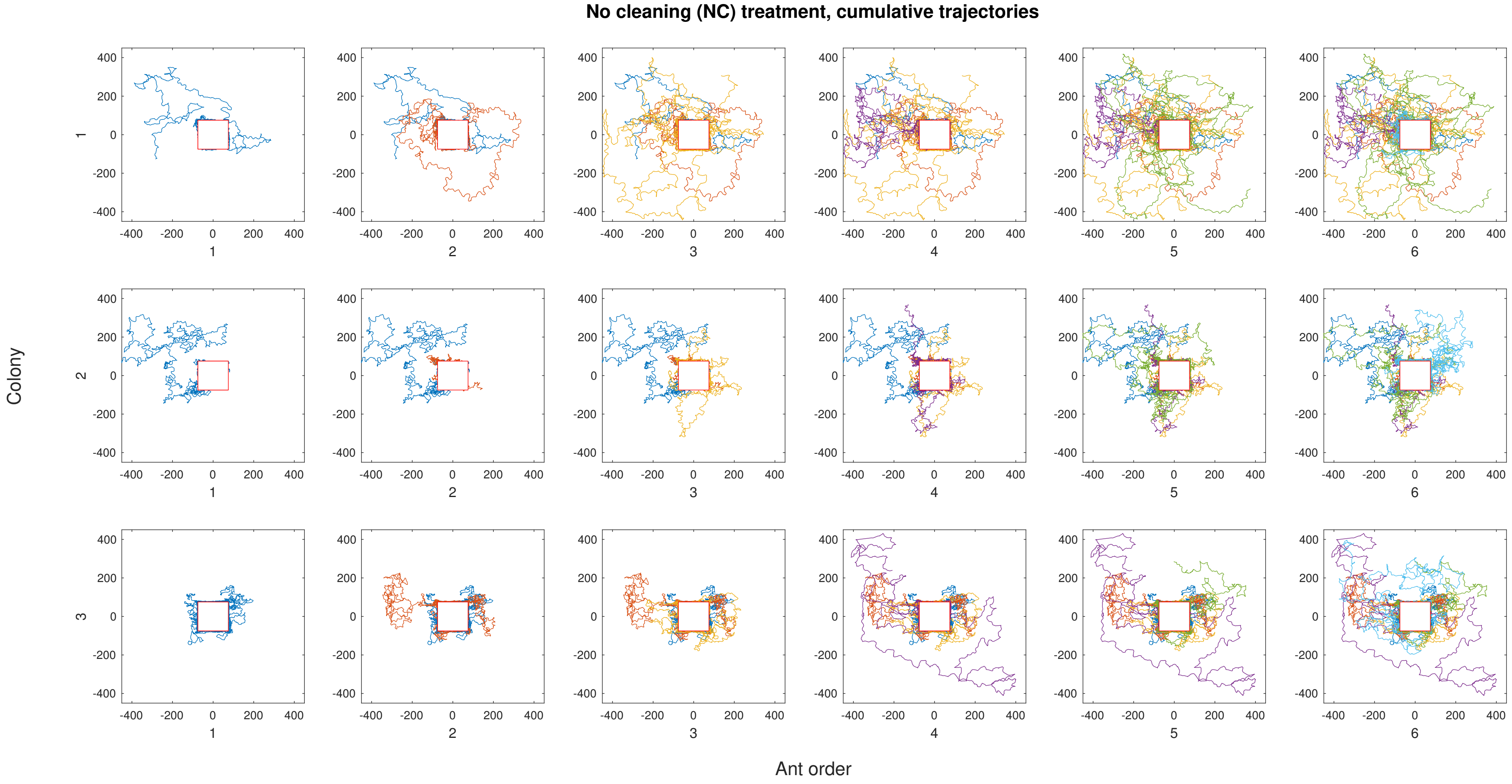

Supplement: Ant trajectories and analysis [file rsif20190848supp1.pdf]
